# Monitoring pilots’ mental workload in real flight conditions using multinomial logistic regression with a ridge estimator

**DOI:** 10.3389/frobt.2025.1441801

**Published:** 2025-04-24

**Authors:** Muhammad Haseeb, Rashid Nadeem, Nazia Sultana, Noman Naseer, Hammad Nazeer, Frédéric Dehais

**Affiliations:** ^1^ Department of Information Engineering, Universitá di Padova, Padova, Italy; ^2^ Department of Electrical and Computer Engineering, Air University, Islamabad, Pakistan; ^3^ Department of Mechatronics and Biomedical Engineering, Air University, Islamabad, Pakistan; ^4^ Institut Supérieur de l’Aéronautique et de l’Espace, Université de Toulouse, Toulouse, France

**Keywords:** dry-electrode EEG, real flight conditions, artifact subspace reconstruction, auditory attention, Enobio neuroelectrics system, passive brain computer interface

## Abstract

Piloting an aircraft is a cognitive task that requires continuous verbal, visual, and auditory attentions (e.g., Air Traffic Control Communication). An increase or decrease in mental workload from a specific level can alter auditory and visual attention, resulting in pilot errors. The objective of this research is to monitor pilots’ mental workload using advanced machine learning techniques to achieve improved accuracy compared to previous studies. Electroencephalogram (EEG) data were recorded from 22 pilots operating under visual flight rules (VFR) conditions using a six dry-electrode Enobio Neuroelectrics system, and the Riemannian artifact subspace reconstruction (rASR) filter was used for data cleaning. An information gain (IG) attribute evaluator was used to select 25 optimal features out of 72 power spectral and statistical extracted features. In this study, 15 classifiers were used for classification. Multinomial logistic regression with a ridge estimator was selected, achieving a significant mean accuracy of 84.6% on the dataset from 17 subjects. Data were initially collected from 22 subjects, but 5 were excluded due to data synchronization issues. This work has several limitations, such as the author did not counter balance the order of scenario, could not control all the variables such as wind conditions, and workload was not stationary in each leg of the flight pattern. This study demonstrates that multinomial logistic regression with a ridge estimator shows significant classification accuracy (p < 0.05) and effectively detects pilot mental workload in real flight scenarios.

## 1 Introduction

Human errors, including pilot errors, are among the major causes of aviation accidents (Li et al., 2001). According to NASA, in 2004, pilot error was listed as the primary cause of 78.6% of fatal general aviation accidents in the United States (Shively, 2013). The International Civil Aviation Organization (ICAO) also states that pilot errors were a contributing factor in 60%–80% of aviation accidents. The Flight Safety Foundation (FSF), another organization that provides information on aviation safety reported in 2020 that human factors such as pilot error, maintenance error, and air traffic control errors contributed to approximately 70% of aviation accidents (Flight Safety Foundation, 2000). According to the Federal Aviation Administration, human factors directly cause or contribute to many aviation accidents and have been documented as a primary contributor in more than 70% of aircraft accidents (Duncan, 2016). In emergency situations, the mental overload experienced by pilots can negatively impact their vision and auditory senses, leading to pilot errors. To combat this issue, the use of a brain–computer interface (BCI) system has been proposed. BCIs enable direct communication between the brain and an external device, such as a computer, without relying on traditional motor output pathways. Among BCIs, a passive brain–computer interface (pBCI) has been developed as a specialized extension designed to monitor mental states such as mental workload (Zander and Kothe, 2011; Aricó et al., 2017). This system utilizes biological signals, such as electroencephalogram (EEG), electrocardiogram (ECG), and eye-tracking signals, to gain insight into the psychological condition of the pilot (Wang et al., 2020). By using portable measurement techniques, the labor-intensive task of data collection is simplified.

**FIGURE 1:**
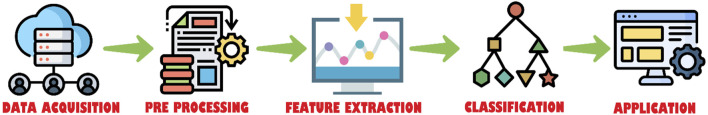
Typical BCI system.

BCIs consist of several components that enable the direct communication of neural signals with external devices, bypassing traditional motor pathways. These components include signal acquisition, signal preprocessing, feature extraction and selection, classification, and application, as shown in Figure 1. Signal acquisition involves collecting neural signals from the user’s brain, followed by preprocessing to enhance data quality. Feature extraction and selection identify relevant information from the signals, which are then classified using machine learning algorithms. The final application component translates these classified outputs into actions, such as controlling external devices or monitoring cognitive states. Passive BCIs, as an extension of BCI, focus specifically on monitoring mental states such as workload without requiring active user engagement. The integration of these components allows pBCIs to provide valuable insights into cognitive states, offering applications in aviation and other high-stakes environments.

Some other authors have also monitored mental workload for different purposes using EEG signals, as shown in Table 1. The feasibility of using EEG in actual flight conditions has been investigated in previous studies (Sauvet et al., 2014; Di Stasi et al., 2015; Sterman et al., 1988; Wilson et al., 1987). However, the EEG system utilized by these authors employed wet electrodes, which require the use of conductive gel on the scalp, making it impractical for daily flight operations. To overcome this limitation, the development of gel-free pre-amplified dry electrodes has been initiated, which also allows for wireless communication protocols (e.g., Wi-Fi and Bluetooth) and provides greater freedom of movement for users during mobile recordings (Blum et al., 2017). Although the use of dry electrodes remains challenging due to their lower signal-to-noise ratio compared to that of wet electrodes (Guger et al., 2012; Searle and measurement, 2000), and several studies have successfully implemented offline pBCIs using dry-electrode EEG systems in actual flight conditions (Dehais et al., 2018; Scholl et al., 2016; Callan et al., 2015). However, the cockpit environment is characterized by high levels of noise from engine vibrations, pilots’ muscular activity, and electromagnetic interference, which can affect the signal-to-noise ratio and, thus, limit the efficacy of dry electrodes. Moreover, the use of multiple channel systems (e.g., 32 or 64 electrodes) in these studies can be cumbersome and uncomfortable for subjects over extended periods of time. A similar approach is to reduce the number of electrodes in the pilots’ headset, but this approach has its own drawback as the reduction in electrodes prevents the use of the independent component analysis (ICA) technique to identify artifactual components (Delorme and Makeig, 2004). Artifact subspace recognition (ASR) is a solution for the abovementioned drawback because it removes short-time high-amplitude artifacts automatically. Recently, Blum et al. (2019) explored Riemannian ASR (rASR), an alternative method for artifact removal with lower computational costs, higher reliability, and greater sensitivity to eye artifacts in mobile EEG data compared to ASR. It is an open-source project and is available as a MATLAB toolbox (rASRMatlab, 2025).

**TABLE 1:** Relative research contributions.

Authors name	No. of channels	Electrode type	Technique type	Objective	Participants	Accuracy
Winnie K. Y. So, Savio W. H. Wong, Joseph N. Mak, Rosa H. M. Chan (So et al., 2017)	1	Dry	EEG	Evaluate dynamic changes in mental workload	University students	65%–75%
Frédéric Dehais, Alban Duprès, Sarah Blum, Nicolas Drougard, Sébastien Scannella, Raphaëlle N. Roy, Fabien Lotte (Dehais et al., 2019)	6	Dry	EEG	Monitoring pilot’s mental workload	Pilots	70%
Jordan J. Bird, Luis J. Manso, Eduardo P. Ribeiro, Anikó Ekárt, Diego R. Faria (Bird et al., 2018)	5	Dry	EEG	Mental state recognition	University students	87%
Adil Deniz DURU (Duru, 2019)	16	Dry	EEG	Determination of increased mental workload condition	University students	96%
Sushil Chandra, Kundan Lal Verma, Greeshma Sharma, Alok Mittal (Chandra et al., 2015)	14	Dry	EEG	Cognitive workload classification	University students	61.68%
Shouyi Wang, Jacek Gwizdka, W. Art Chaovalitwongse (Wang et al., 2016)	14	Dry	EEG	Using wireless EEG signals to assess memory workload	University students	81% (Entire session using all trials)
Sanay Muhammad Umar Saeed, Syed Muhammad Anwar, Muhammad Majid, Muhammad Awais, Majdi Alnowami (Saeed et al., 2018)	1	Dry	EEG	Human stress classification	University students or faculty member	83.33%
Gerald Matthews, Lauren Reinerman-Jones, Julian Abich IV, Almira Kustubayeva (Matthews et al., 2017)	9	Dry	EEG	To compare alternate metrics of EEG response to cognitively Demanding tasks as indicators of operator functional status	University students	80.3% (Threat detection)
Hongquan Qu, Yiping Shan, Yuzhe Liu, Liping Pang, Zhanli Fan, Jie Zhang, Xiaoru Wanyan (Qu et al., 2020)	32	Dry	EEG	Mental workload classification method based on EEG independent component features	University students	79.8% (Method 1)
Hamed Taheri Gorji, Nicholas Wilson, Jessica VanBree, Bradley Hoffmann, Thomas Petros (Taheri Gorji et al., 2023)	20	Dry	EEG	Discriminate the aircraft pilot cognitive workload during flight	Collegiate aviation students	91.67%
Salvan L, Paul TS, Marois A (32)	3	Dry	EEG	Dry EEG-based mental workload prediction for aviation	Random participants	76%
Wang Y, Han M, Peng Y, Zhao R, Fan D, Meng X (Blum et al., 2019)	32	Dry	EEG	Learning local–global EEG representations for cognitive workload classification in simulated flights	Random participants	91.19%
Ibrahim Alreshidi, Irene Moulitsas, Karl W. Jenkins (Alreshidi et al., 2023)	20	Dry	EEG	Mental state detection	Pilots	86%
Bas Verkennis, Evy van Weelden, Francesca L. Marogna, Maryam Alimardani, Travis J. Wiltshire, Max M. Louwerse (Verkennis et al., 2024)	14	Dry	EEG	Mental workload prediction	Pilots (virtual flight simulation)	78%
Hernández-Sabaté A, Yauri J, Folch P, Piera MÀ, Gil D (Hernández-Sabaté et al., 2022)	14	Dry	EEG	Recognition of the mental workloads	Pilots (in cockpit)	82.03%
Lee DH, Kim SJ, Kim SH, Lee SW (Lee et al., 2024)	34	Dry	EEG + EOG	Decoding EEG–based workload levels	Pilots (in simulator)	86.13%

EEG data are crucial for monitoring and assessing mental workload by analyzing neural activity patterns (Dan and Reiner, 2017). Alpha, beta, theta, gamma, and delta waves in EEG signals have different frequencies and amplitudes, providing insights into various cognitive states (Harmony, 2013). The higher frequency and lower amplitude of beta waves mostly relate to higher activity in the brain (Schmidt et al., 2019), whereas the higher amplitude alpha waves diminish when the brain is hyperactive (Klimesch, 1999). Gamma waves indicate voluntary motor movement and learning processes (Ulloa, 2022). Research related to neuroscience considers non-invasive and invasive EEG recordings to be the candidate techniques for BCI applications. Invasive techniques require electrode implantation in the cortex, and recording EEG is a time-consuming process, which might result in medical complications. On the other hand, non-invasive EEG methods avoid such invasive procedures, reducing the likelihood of complications (Posada-Quintero et al., 2019). Experiments consisting of low- and high-load conditions were carried out by Soeiro (2019). In low-load conditions, the pilots observed, while the flight instructor handled the flight, whereas in high-load conditions, they operated the plane themselves. The analysis showed higher band power for theta and alpha in the low-load condition, and 70% classification accuracy was achieved through extracted frequency features (Soeiro, 2019).

Recent studies have used classifiers to predict outcomes; an example is linear regression with a ridge estimator. The studies show the use of regression analysis on predictors based on EEG and heart rate to test the activeness of the brain while facing obstacles (Kabir et al., 2016). Logistic regression (LR) is a widely used method for classifying binary data. It aims to classify a dataset into a categorical variable or binary using the logistic regression function. LR is a specialized form of regression used to classify data of an event according to Bernoulli distribution. Previous studies often relied on EEG data collected under controlled settings, which may not fully represent the noisy environment of the cockpit (Wang et al., 2024). Advancements in technology have facilitated the retrieval of EEG signals during the actual flight operations. Dry EEG systems, such as the six-electrode system used in this study, are easier to wear and are more practical for operational environments. However, their lower signal-to-noise ratio can impact classification accuracy, particularly in noisy cockpit environments, influenced by vibrations and electromagnetic interferences (Lujan-Moreno et al., 2014). In a prior study that utilized three dry electrodes (Salvan et al., 2023), the accuracy was 76%. To improve the accuracy, this study incorporates six dry electrodes, thereby enhancing the richness and depth of data collection. This underscores the robustness and effectiveness of assessing pilots’ mental workload in real flight scenarios, thereby contributing to enhanced aviation safety measures.

This study proposes a mechanism that can minimize human error and enhance passenger safety by monitoring the pilot’s mental condition. This study processes data from a previous study (Dehais et al., 2019). The data have already been preprocessed (Dehais et al., 2019), and the focus of this study is to enhance the accuracy using advanced machine learning techniques. In addition to power features, statistical features were also extracted to increase the accuracy. To optimize processing speed, the significant features were selected using the information gain feature selection method, as shown in Figure 12, implemented by WEKA. Various machine learning classification models, such as naïve Bayes, linear regression, logistic regression, random forest, and decision tree, were studied and applied sequentially using WEKA to achieve enhanced accuracy, as demonstrated in Figures 10, 11.

This study introduces significant advancements in monitoring pilots’ mental workload in real flight scenarios. Unlike many prior studies that relied on cumbersome EEG setups with multiple electrodes (e.g., 16 or more), this research employs a six-dry-electrode EEG system, overcoming these limitations and offering a practical, user-friendly solution suitable for operational settings. Additionally, the study is conducted under real flight conditions with pilots as participants, addressing challenges such as cockpit noise, vibrations, and electromagnetic interference. These factors make the findings directly applicable to aviation safety, whereas many previous studies relied on university students or pilots in controlled laboratory environments. The incorporation of both power and statistical features enhances the richness of the data, while advanced feature selection through the information gain method and classification using multinomial logistic regression with a ridge estimator achieves a significant accuracy of 84.6%. The study also emphasizes the importance of balancing accuracy and computational efficiency, identifying a feature set that optimally reduces classification time to just 0.03 s without compromising the accuracy. Furthermore, statistical validation through a Student’s t-test confirms the superiority of the proposed classifier, ensuring reliability and reproducibility. By achieving competitive accuracy with fewer electrodes, this research sets a new benchmark for simplified and effective EEG-based monitoring systems in aviation, demonstrating its potential to enhance safety and reduce human error.

## 2 Materials and methods

### 2.1 Material

**FIGURE 2:**
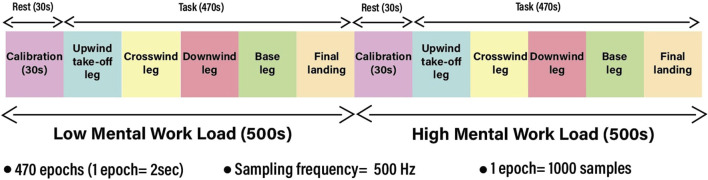
Flight scenario.

**FIGURE 3:**
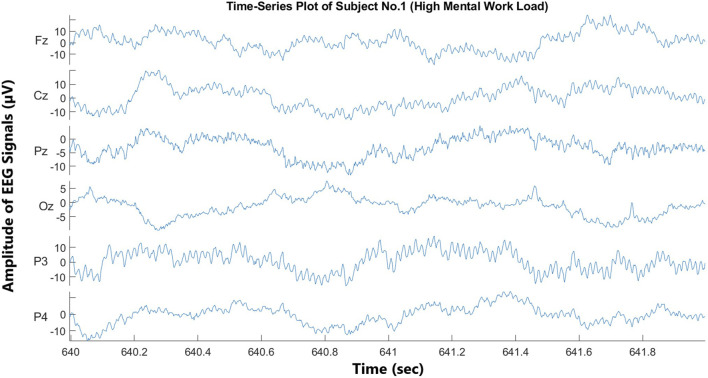
Time-series plot of EEG signals under high-mental workload (subject no. 1).

**FIGURE 4:**
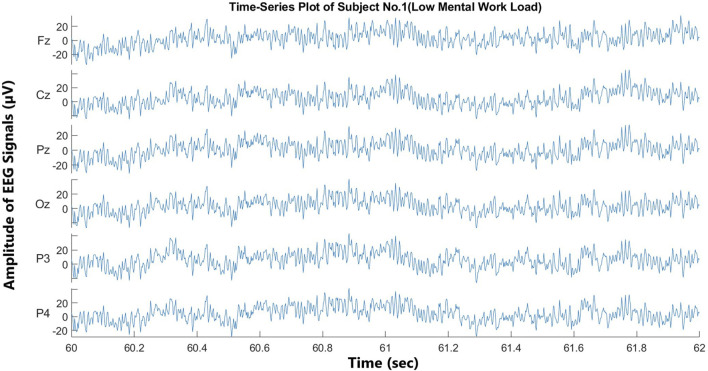
Time-series plot of EEG signals under low-mental workload (subject no. 1).

Twenty-two pilots operating under visual flight rules (VFR) completed the experiment, and all of them passed the medical fitness test for flying. In the previous study, four subjects were excluded due to data synchronization issues (Dehais et al., 2019). However, during the preprocessing phase of this study, subject number 17 was identified with severe EEG signal inconsistencies that were not previously detected. These inconsistencies were observed during feature extraction and classification, leading to the exclusion of this subject to ensure data reliability. As a result, a total of five subjects were rejected in this study due to data synchronization issues. The total duration of a subject’s session was approximately 1 hour. The study was conducted using the ISAE-SUPAERO experimental light aircraft, and the DR400 light aircraft was powered by a 180 HP Lycoming engine. The flight scenario consisted of two consecutive traffic patterns, each divided into five flight phases according to VFR (Figure 2). In the first traffic pattern, i.e., the low-load condition, the participant (left-seated) monitored the flight controlled by the flight instructor (right-seated). In the second traffic pattern, i.e., the high-load condition, the participant was flying the aircraft under the supervision of the flight instructor. Time series plots (2-second duration) of all the channels of subject no. 1 under high- and low-load conditions are shown in Figures 3, 4, respectively. The EEG data were divided into successive, non-overlapping epochs of 2 s, independent of stimuli onset. Each epoch contained 1,000 samples (with a sampling frequency of 500 Hz). This segmentation method was chosen to ensure a continuous representation of the pilot’s mental workload during the flight, rather than being tied to specific task events. Each traffic pattern lasted approximately 500 s, and the total experiment duration was approximately 20 min, covering the full flight process from takeoff to parking. Prefatory experiments were conducted with four pilots to pre-test the experimental scenario. NASA-TLX score confirmed two different levels of mental workload (high-load condition = 6.7, SD0.45; low-load condition 2.56, SD = 0.75) based on two elicited conditions (Dehais et al., 2019).

**FIGURE 5:**
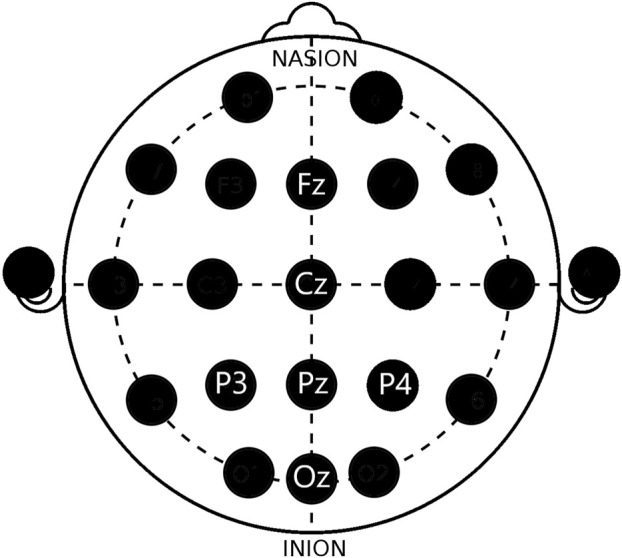
Electrode positioning (10–20 system).

EEG data were recorded using six dry-electrodes from the Enobio Neuroelectrics system (Fz, Cz, Pz, Oz, P3, and P4 sites) positioned according to the 10–20 system at 500 Hz, as shown in Figure 5. DRL and CMS were used as reference electrodes (Dehais et al., 2019). For rASR calibration, cleaned data were used, and all EEG analyses were run using EEGLAB (V14.1.2) and MATLAB (). In data preprocessing, frequency domain analysis was used, where data were high-pass (0.5 Hz) filtered and then processed using the rASR plugin to remove noise (Dehais et al., 2019). Noisy portions of data (e.g., trials) were cleaned using the Riemannian ASR (rASR) version of the clean raw data MATLAB toolbox. The toolbox contains the core functionality clean_asr to correct data segments that can be applied if short parts of the data are artifactual or only a minor portion of all channels is affected. Parameters used for clean_asr were as follows: flatline criterion = 5, highpass = [0.25 0.75], channel criterion = 0.85, line noise criterion = 4, burst criterion = 70, and window criterion = 0.10. The experiment was approved by the European Aviation Safety Agency (EASA60049235). The methods were carried out in accordance with approved guidelines, and participants provided their informed written consent.

### 2.2 Methods

#### 2.2.1 Feature extraction

Feature extraction includes extracting the features, also known as input attributes, that will be used in the classification of mental workload. Following the literature review (Dan and Reiner, 2017; So et al., 2017; Duru, 2019), it is observed that the main feature for mental workload classification is the band power of delta, theta, alpha, beta, and gamma channels. For six channels, the extracted power features (band power of delta, theta, alpha, beta, and gamma x 6) are 30, and some statistical features, which include mean, standard deviation, maximum, variance, area under the curve, skewness, and kurtosis, were also used. The total number of extracted temporal and spectral features was 72, including both power and statistical features. The statistical features were calculated using MATLAB’s built-in functions for efficiency and accuracy. These features included mean (mean), standard deviation (std), maximum (max), the area under the curve (trapz), kurtosis (kurtosis), variance (var), and skewness (skewness). These computations were applied to the amplitude time series of EEG signals from each channel to extract relevant features for classification.

#### 2.2.2 Feature selection

In machine learning and statistics, feature selection, also known as variable selection, attribute selection, or variable subset selection, is the process of selecting a subset of relevant features for use in model construction. Feature selection techniques are used for several reasons, which include simplifying models to enhance interpretability for researchers/users (James et al., 2013), reducing training times, avoiding the curse of dimensionality, and improving generalization by reducing overfitting (Bermingham et al., 2015) [formally, reduction of variance (Bermingham et al., 2015)]. In this study, the objective of feature selection is three-fold, which includes improving the prediction performance of the predictors, providing faster and more cost-effective predictors, and providing a better understanding of the underlying process that generated the data (Guyon and research AEJ of machine learning, 2003).

For feature selection, the information gain attribute evaluator (InfoGainAttributeEval) and correlation attribute evaluator (CfsSubsetEval) were used to compare the optimal evaluator with nine-fold cross validation. Information gain evaluates the relevance of each feature by measuring the reduction in entropy when a feature is included in the model, while the correlation-based feature selection method selects the subsets of attributes that are highly correlated with the class variable but exhibit minimal correlation with each other, reducing redundancy. The Ranker search method (weka.attributeSelection.Ranker) was applied to rank features individually. The Ranker search was configured to generate ranking (generateRanking = True), ensuring that the attributes were ranked based on their individual evaluation scores. All features were initially considered for ranking (numToSelect = −1), meaning that no attributes were removed unless explicitly set by a threshold. The threshold was left at its default value (−1.7976931348623157E308), ensuring that no attributes were discarded unless they contributed negligibly to classification performance. The startSet parameter was left empty to allow the evaluation of all features during the ranking process. Multinomial logistic regression was used as a fitness model benchmark to select the optimal attribute evaluator. The InfoGain attribute evaluator with 25 features, as shown in Figure 12, was selected as the optimal evaluator, as shown in Tables 2, 3. Feature selection and classification were performed using an Intel Core i5-4210U CPU @ 1.70 GHz with 4 GB RAM and implemented using WEKA (Partners, 2020).

**TABLE 2:** Subject-specific classification performance using information gain-based feature selection with nine-fold cross-validation.

Subject No.	40 features		35 features		30 features		25 features		20 features	
	Time (Sec)	Accuracy %	Time (Sec)	Accuracy %	Time (Sec)	Accuracy %	Time (Sec)	Accuracy %	Time (Sec)	Accuracy %
1	0.3	94.68	0.05	95.53	0.05	95.10	0.03	96.81	0.03	97.02
2	0.06	87.66	0.05	87.02	0.04	85.74	0.06	86.38	0.03	85.53
3	0.04	85.53	0.04	85.10	0.03	82.13	0.02	83.40	0.01	82.77
4	0.1	82.55	0.07	82.77	0.04	82.77	0.04	83.19	0.03	82.34
5	0.15	82.77	0.07	81.70	0.06	82.34	0.03	83.62	0.02	76.81
6	0.1	63.19	0.02	56.38	0.02	61.70	0.01	60.00	0.01	56.60
7	0.07	85.96	0.04	86.60	0.05	84.47	0.02	84.68	0.02	83.19
8	0.05	87.23	0.04	88.30	0.04	88.94	0.03	88.94	0.03	86.17
9	0.11	90.43	0.07	91.49	0.06	91.06	0.03	90.85	0.02	91.06
10	0.09	82.77	0.05	82.77	0.07	82.98	0.03	83.83	0.03	83.19
11	0.07	96.38	0.06	96.60	0.05	97.23	0.02	98.08	0.04	97.23
12	0.02	75.74	0.01	75.96	0.01	76.17	0.01	74.68	0.01	74.25
13	0.41	92.76	0.2	93.62	0.12	93.61	0.08	94.25	0.04	94.89
14	0.03	71.27	0.03	70.64	0.03	71.70	0.02	71.06	0.02	71.06
15	0.33	91.70	0.18	93.19	0.04	92.34	0.03	92.76	0.02	92.76
16	0.03	77.23	0.01	73.40	0.01	72.34	0.01	71.91	0.01	71.06
18	0.09	92.13	0.05	92.77	0.04	94.04	0.04	93.83	0.03	94.47
Mean	0.121	84.70	0.06	84.34	0.05	84.39	0.03	84.6	0.02	83.55
Std	0.11	8.82	0.05	10.49	0.02	9.65	0.01	9.97	0.01	11.01

**TABLE 3:** Subject-specific classification performance using correlation-based feature selection with nine-fold cross-validation.

Subject No.	40 features		35 features		30 features		25 features		20 features	
	Time (Sec)	Accuracy %	Time (Sec)	Accuracy %	Time (Sec)	Accuracy %	Time (Sec)	Accuracy %	Time (Sec)	Accuracy %
1	0.29	94.68	0.08	95.53	0.03	96.38	0.02	97.23	0.03	96.80
2	0.11	88.93	0.04	88.94	0.05	87.87	0.03	87.65	0.03	87.87
3	0.09	94.89	0.04	96.17	0.04	95.74	0.03	79.57	0.01	78.51
4	0.06	81.06	0.04	81.28	0.04	81.49	0.03	80.64	0.01	79.36
5	0.08	85.74	0.05	85.95	0.04	84.04	0.02	81.70	0.02	82.98
6	0.03	89.15	0.03	89.15	0.02	66.60	0.01	62.77	0.01	61.28
7	0.04	84.47	0.04	84.47	0.03	82.97	0.01	82.55	0.05	81.70
8	0.03	67.45	0.03	67.65	0.03	68.30	0.02	61.91	0.01	61.91
9	0.1	90.64	0.09	89.78	0.04	89.36	0.03	90.21	0.02	89.79
10	0.05	85.32	0.05	84.68	0.04	85.32	0.04	85.32	0.03	83.40
11	0.09	95.32	0.09	95.96	0.08	97.23	0.04	98.30	0.03	98.51
12	0.03	75.11	0.02	74.89	0.02	76.38	0.01	76.38	0.01	74.89
13	2.95	92.34	0.36	93.19	0.17	93.40	0.05	93.83	0.03	94.47
14	0.03	70.64	0.03	68.30	0.02	67.66	0.01	69.15	0.01	67.02
15	0.11	93.19	0.05	93.62	0.04	93.83	0.04	93.83	0.03	92.98
16	0.02	72.12	0.02	72.13	0.01	72.13	0.01	70.21	0.02	70.43
18	0.08	91.49	0.14	91.91	0.04	93.40	0.11	93.19	0.03	93.83
Mean	0.25	85.44	0.07	85.5	0.04	84.24	0.03	82.61	0.02	82.102
Std	0.69	9.07	0.08	9.54	0.03	10.62	0.02	11.52	0.01	11.90

##### 2.2.2.1 Information gain attribute evaluator

Information gain (IG) shows how much an attribute contributes to predicting the output by measuring the reduction in entropy. Its value varies from 0 to 1, where 0 shows no information, meaning that the attribute can be removed from the dataset, while 1 shows that this attribute plays a maximal role in predicting the output. WEKA (Partners, 2020) supports feature selection via information gain using the InfoGainAttributeEval attribute evaluator with the ranker search method.

The entropy of Y is shown in Equation 1 as follows:

$$H\left(Y\right)=-\sum_{y\in Y}p\left(y\right)\log_{2}\left(p\left(y\right)\right),$$ (1)

where p(y) is the marginal probability density function for the random variable Y. Then, the entropy of Y after observing X is shown in Equation 2 as follows:

$$H\left(\frac{Y}{X}\right)=-\sum_{x\in X}p\left(x\right)\sum_{y\in Y}p\left(\frac{y}{x}\right)\log_{2}\left(p\left(\frac{y}{x}\right)\right),$$ (2)

where p (y |x) is the conditional probability of y given x. Given the entropy is a criterion of impurity in a training set S, we can define a measure reflecting additional information on Y provided by X that represents the amount by which the entropy of Y decreases. This measure is known as IG. It is given by the formula for IG is shown in Equation 3 as follows

$$IG=H\left(Y\right)-H\left(\frac{Y}{X}\right)=H\left(X\right)-H\left(\frac{X}{Y}\right).$$ (3)

The information gained on Y after observing X is equal to the information gained on X after observing Y. A weakness of the IG criterion is that it is biased in favor of features with more values even when they are not more informative (TELFOR JN, 2009).

##### 2.2.2.2 Correlation attribute evaluator

Correlation shows the relationship between features and the target variable. The optimal features are those that are more related to the targeted variable. Correlation can be positive (an increase in one value of the feature increases the value of the target variable) or negative (an increase in one value of the feature decreases the value of the target variable). The correlation attribute evaluator (CAE) evaluates subsets of features on the basis of the following hypothesis: “good feature subsets contain features highly correlated with the classification, yet uncorrelated to each other” (Hall, 1999; Senliol and Gulgezen, 2014). The merit of a feature subset S consisting of k features is shown in Equation 4 as follows:

$${Merit}_{s_{k}}=\frac{k\bar{r_{cf}}}{\sqrt{k+k\left(k-1\right)\bar{r_{ff}}}}.$$ (4)

Here, $\bar{rcf}$ is the average value of all feature–classification correlations, and $\bar{rff}$ is the average value of all feature–feature correlations. The CFS criterion is shown in Equation 5 as follows:

$$CFS=\max_{S_{k}}\left[\;\frac{r_{cf_{1}}+r_{cf_{2}}+\cdot \cdot \cdot r_{cf_{k}}}{\sqrt{k+2\left(r_{f_{1}f_{2}}+\cdot \cdot \cdot r_{f_{i}f_{j}}+\cdot \cdot \cdot r_{f_{k}f_{k-1}}\right)}}\right].$$ (5)

#### 2.2.3 Classification

In machine learning, the classification technique is used to distinguish between two or more than two classes. For mental workload detection, 15 classifiers have been used in this study, as shown in Figures 10, 11, and the maximum accuracy has been achieved by multinomial logistic regression with a ridge estimator implemented by WEKA. To determine the best-performing classifier for this study, multiple machine learning algorithms are applied and evaluated using the WEKA tool. These algorithms included naïve Bayes, multinomial logistic regression, multilayer perceptron, simple logistic regression, SMO, decision trees, and several others. The performance of each classifier was assessed based on its mean classification accuracy from nine-fold cross validation, as depicted in Figure 10. Among the tested algorithms, multinomial logistic regression emerged as the best-performing classifier, achieving the highest mean accuracy of 84.6%, as shown in Figure 10. This methodology ensured an objective comparison by selecting the classifier that demonstrated the most reliable and accurate predictions for the dataset.

This classifier extends traditional logistic regression by incorporating a ridge penalty (ridge = 1.0E-8) to mitigate overfitting while maintaining model stability. The optimization of model parameters was performed using the quasi-Newton method, which is well-suited for handling high-dimensional datasets. Missing values within the dataset were handled using WEKA’s built-in ReplaceMissingValuesFilter, preventing inconsistencies in the training process. Additionally, the classifier was configured with a batch size of 100 to optimize processing efficiency. The maximum number of iterations (maxIts) was set to −1, allowing the model to iterate until convergence. The doNotCheckCapabilities setting was kept at false, ensuring that the classifier constraints were validated before execution to maintain consistency in model training and evaluation.

Standardization or normalization prevents potential biases from certain attributes within the dataset. In this study, no explicit normalization or standardization of the feature sets was performed prior to training the algorithms. This approach leverages the preprocessing capabilities of the WEKA tool, which ensures that if the instance weights are not uniform, the data are resampled with replacement based on the weights before being passed to the base classifier. Therefore, WEKA served the purpose and prevented potential biases from certain attributes within the dataset.

For classification, the authors used nine-fold cross validation to evaluate the model performance. The dataset was divided into nine subsets, with each subset serving as the test set once, while the remaining eight subsets were used for training. Model parameters were also finalized based on cross-validation performance using a nine-fold cross-validation approach. This ensured that the selected parameters generalize well to unseen data while minimizing overfitting. Additionally, default WEKA settings were retained for hyperparameters where prior experimentation indicated stability.

##### 2.2.3.1 Multinomial logistic regression

Multinomial logistic regression is one of the most important classifiers for analyzing categorical data (El-Habil, 2012). This model deals with one nominal/ordinal response variable that has equal or more than two categories, whether it is a nominal or ordinal variable. This model has been applied to data analysis in many areas, including health, social sciences, behavioral studies, and education (El-Habil, 2012). By using this classifier, the mean classification accuracy of 84.6% and mean classification time of 0.03 s were achieved, and the remarkable accuracy among all of them is shown in Figures 10, 11.

## 3 Results

### 3.1 Feature selection results

**FIGURE 6:**
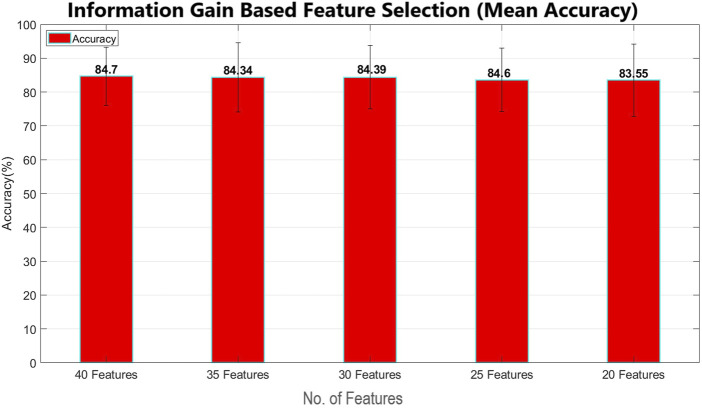
Mean classification accuracy using multinomial logistic regression (cross validation = 9 folds) for information gain-based feature selection (error bars represent standard deviation).

**FIGURE 7:**
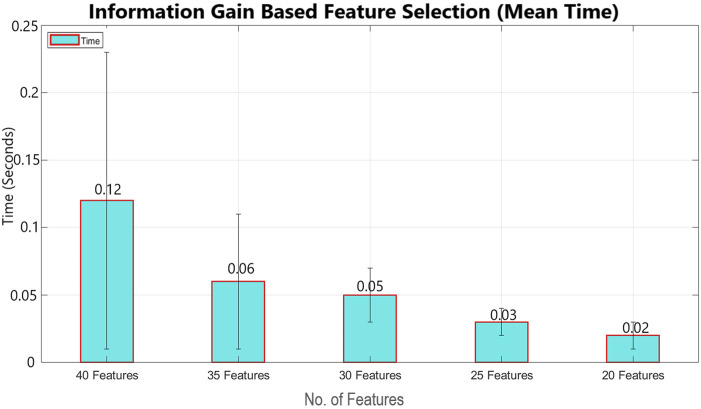
Mean classification time using multinomial logistic regression (cross validation = 9 folds) for information gain-based feature selection (error bars represent standard deviation).

**FIGURE 8:**
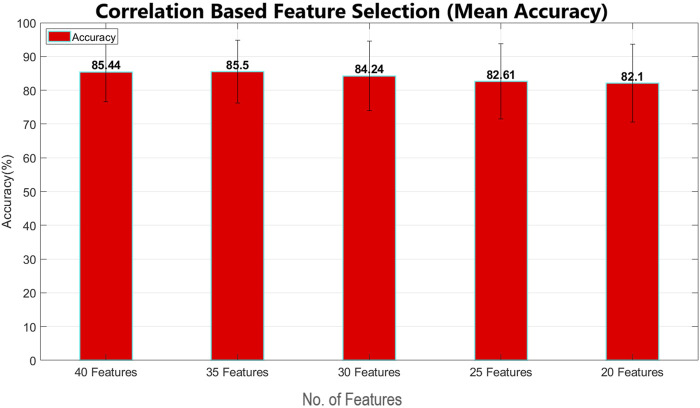
Mean classification accuracy using multinomial logistic regression (cross validation = 9 folds) for correlation-based feature selection (error bars represent standard deviation).

**FIGURE 9:**
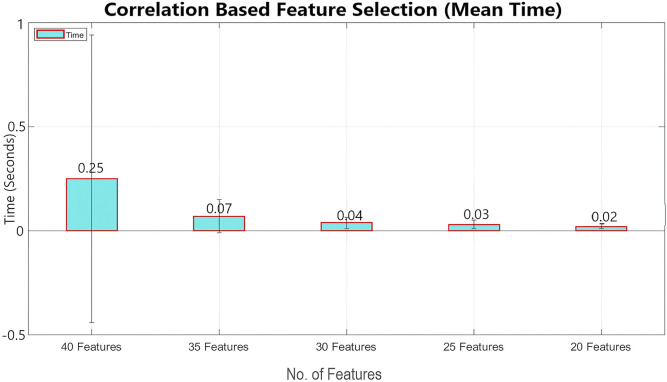
Mean classification time using multinomial logistic regression (cross validation = 9 folds) for correlation-based feature selection (error bars represent standard deviation).

Feature selection has been performed using two main feature-selection algorithms known as information gain and correlation implemented by WEKA. Multinomial logistic regression with a ridge estimator has been used as a benchmark classifier to select the best feature selection method. For this purpose, the model has been classified using 40 features, 35 features, 30 features, 25 features, and 20 features, with both feature selection methods, as shown in Tables 2, 3. Mean classification time and accuracy are shown in Figures 6–9. Correlation-based feature selection with 35 features shows the highest mean accuracy of 85.5%, while correlation-based feature selection with 20 features shows a mean accuracy of 82.1%, as shown in Figures 8, 9. It is concluded that when the number of features decreases, the accuracy also decreases, and the classification time also reduces. To monitor the pilot’s mental workload, the classification time should be as low as possible, so in terms of classification, time correlation-based feature selection with 35 features is not a preferred solution. A more efficient solution is the one with high accuracy and less classification time, so it is a tradeoff between accuracy and time. Information gain-based feature selection with 25 features shows a mean accuracy of 84.6% and a mean classification time of 0.03 s, as shown in Figures 6, 7, which is a preferred solution. The EEG signal of each subject differs from that of the others, which leads to variations in the best-selected features for each subject, as shown in Figure 12. However, the percentage distribution of features remains consistent across subjects, with an average of approximately 50% power-based features and 50% statistical features being selected.

The IG and CAE feature selection methods have key differences in terms of mean accuracy and computation time. IG demonstrates better stability in accuracy across varying feature subsets, maintaining a consistent performance as the number of features decreases. The accuracy of IG decreases from 84.7% for 40 features to 83.55% for 20 features. In contrast, CAE starts with higher accuracy for larger feature sets, achieving 85.44% for 40 features, but accuracy decreases significantly as the number of features decreases, reaching 82.1% for 20 features. Moreover, regarding computation time, IG is faster, taking only 0.12 s for 40 features and 0.03 s for 25 features, while CAE requires significantly more time for larger feature subsets, such as 0.25 s for 40 features. This makes IG more efficient, especially in time-sensitive applications.

For real-time classification tasks such as monitoring the pilot’s mental workload, minimizing classification time is critical. CAE with 35 features, despite offering higher accuracy, is not a preferred solution due to its relatively longer classification time. The best solution lies in achieving a balance between accuracy and computation time. IG with 25 features, as shown in Figure 12, achieves a mean accuracy of 84.6% and a mean classification time of just 0.03 s, making it a more suitable choice for real-time applications. This tradeoff between accuracy and time shows the importance of selecting a method that ensures significant accuracy while maintaining low classification time.

### 3.2 Classifier selection results

**FIGURE 10:**
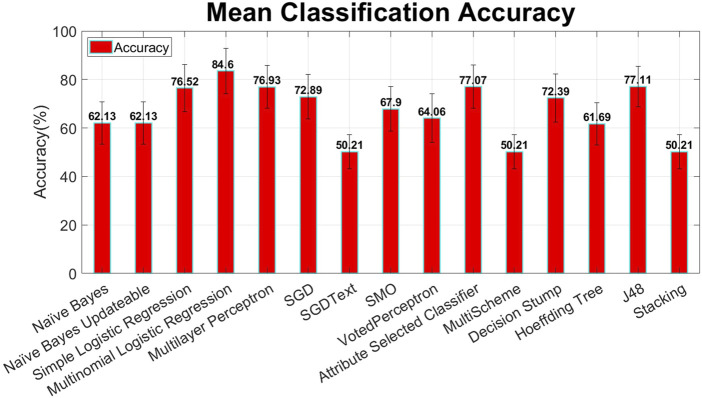
Mean classification accuracy (cross validation = 9 folds) of different classifiers without feature selection (error bars represent standard deviation).

**FIGURE 11:**
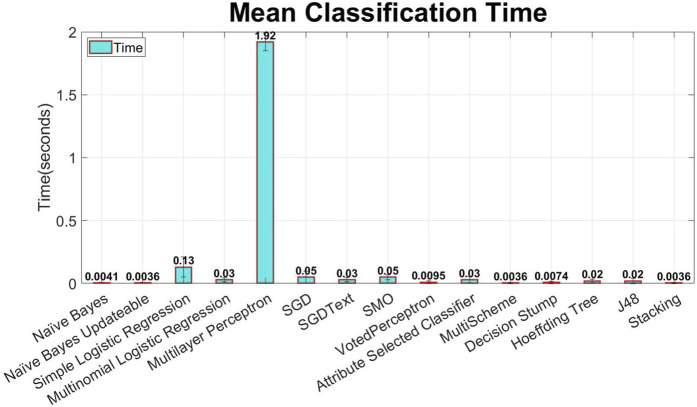
Mean classification time (cross validation = 9 folds) of different classifiers without feature selection (error bars represent standard deviation).

**FIGURE 12:**
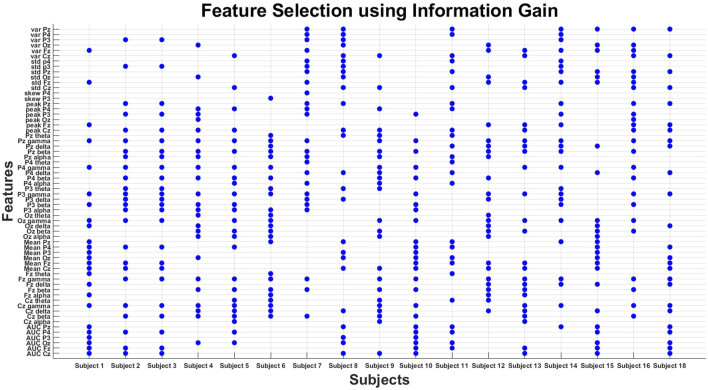
Best features of all subjects using information gain.

Fifteen classifiers, namely, naïve Bayes, naïve Bayes updateable, simple logistic regression, multinomial logistic regression, multilayer perceptron, SGD, SGDText, SMO, VotedPerceptron, attribute-selected classifier, MultiScheme, decision stump, Hoeffding tree, J48, and stacking, were used to select the optimal classifier, as shown in Figures 10, 11. Information gain-based feature selection with 25 features has been used as a benchmark to select the optimal classifier. Multinomial logistic regression with a ridge estimator shows the significant mean classification accuracy from nine-fold cross validation of 84.6% (mean precision = 85.07% and mean recall = 84.6%) and a mean classification time of 0.03 s, as shown in Figures 10, 11. The study shows that statistical features can also be used along with power features, as shown in Figure 12, to increase the classification accuracy and decrease the computational cost and time; the information gain feature selection performs better. For classification purposes, multinomial logistic regression with a ridge estimator shows the optimal solution, as shown in Figures 10, 11. The null hypothesis/significant test was also performed using the Student’s t-test (two tailed distribution), as shown in Table 4. The Student’s t-test with a two-tailed distribution was performed to statistically compare the classification performance of multinomial logistic regression with that of other classifiers. This test used the accuracy results from multiple classification runs to determine whether the observed differences in performance were statistically significant or occurred by chance. The results show that multinomial logistic regression significantly outperformed classifiers such as naïve Bayes and naïve Bayes updateable (p-values = 1.42971E−07), providing strong statistical evidence of its superior performance. This analysis validates the observed differences in accuracy, reinforcing the conclusion that multinomial logistic regression is a more effective classifier in this context.

**TABLE 4:** Student t-test (independent) with two-tailed distribution of multinomial logistic regression classifier vs. the other classifiers (p-value <0.05).

Classifier	p-value	t-statistic
Naïve Bayes	1.42971E-07	6.70
Naïve Bayes updateable	1.42971E-07	6.70
Simple logistic regression	0.045126165	2.08
Multilayer perceptron	0.047	2.06
SGD	0.002686	3.25
SGDText	2.13217E-15	14.23
SMO	3.80618E-05	4.77
VotedPerceptron	2.68812E-06	5.68
Attribute selected classifier	0.052322881	2.01
MultiScheme	2.13217E-15	14.23
Decision stump	0.002548924	3.27
Hoeffding tree	9.90234E-08	6.83
J48	0.048	2.05
Stacking	2.13217E-15	14.23

## 4 Discussion

In actual flight conditions (Sauvet et al., 2014; Di Stasi et al., 2015; Sterman et al., 1988), EEG has been tested by the pioneering work of Wilson et al. (1987). However, these authors used a wet-electrode-based EEG system that might not be feasible for daily flight operations due to the use of conductive gel on the user’s scalp. With advancements in technology, the development of gel-free, pre-amplified dry electrodes has started. Furthermore, the use of wireless communication protocols (e.g., Wi-Fi and Bluetooth) provides freedom of movement for a user and enables signal processing during mobile recordings (Blum et al., 2017). As classical wet/gel electrodes have a higher signal-to-noise ratio than dry electrodes, the use of dry electrodes remains challenging (Guger et al., 2012; Searle and measurement, 2000). The cockpit environment is particularly noisy due to vibrations (e.g., engine), pilots’ muscular activity, and electromagnetic interferences, so the signal-to-noise ratio issue might be critical. Despite all these technical challenges, some authors tested dry-electrode EEG systems in actual flight conditions and implemented offline pBCIs successfully (Dehais et al., 2018; Scholl et al., 2016; Callan et al., 2015). In these studies, multiple channel systems (32 or 64 electrodes) were used, which are cumbersome and cannot be worn by subjects for long periods of time. A similar approach is to reduce the number of electrodes in the pilots’ headset, but this approach has the drawback of preventing the use of the ICA technique to identify artifactual components (Delorme and Makeig, 2004).

This study employs a novel approach to monitor pilots’ mental workload using a six-dry-electrode EEG system in real flight conditions. Previously, Taheri Gorji et al. (2023) achieved 91.67% accuracy with a 20-electrode system, demonstrating the potential of advanced setups, but it was at the cost of increased complexity and reduced practicality. Similarly, Matthews et al. (2017) reported an accuracy of 80.3% using nine electrodes for cognitive task detection, and Wang et al. (2016) achieved 81% accuracy with 14 electrodes. The approach in this study outperforms both while utilizing fewer electrodes. A recent study using 32 electrodes achieved commendable accuracies of 91.19% based on cross-clip data and 83.26% based on cross-session data (Blum et al., 2019), and this study demonstrates competitive accuracy with six electrodes, reaching 84.6% accuracy. This achievement showcases the efficacy of this approach in accurately assessing pilot mental workload during real flight conditions. By leveraging advanced machine learning techniques and optimizing feature selection, this study sets a new benchmark for cognitive workload assessment in aviation, emphasizing the importance of simplicity and effectiveness in EEG-based monitoring systems.

In this study, data from a previous paper (Dehais et al., 2019) were used. The proposed data were already preprocessed (Dehais et al., 2019), so the main objective of this study was to apply advanced machine learning techniques to enhance classification accuracy. Other than power features, statistical features have been used to increase the accuracy, as shown in Figure 12. In order to optimize the processing speed, the most relevant features have been selected using information gain. Multiple classification models of machine learning, such as naïve Bayes, linear regression, logistic regression, random forest, and decision tree, have been studied and applied one by one in order to obtain significant accuracy, as shown in Figures 10, 11. Considering the same scenario—using a six-channel dry electrode EEG system to monitor pilot mental workload in real flight conditions—this study achieves the highest reported accuracy of 84.6% to the best of the author’s knowledge.

This study achieved better accuracy compared to previous studies due to several reasons. First, the author extracted a total of 72 features, including power and statistical features, using MATLAB, which allowed for a more comprehensive analysis of the data. Second, the careful selection of relevant features and the use of appropriate classification techniques further improved the accuracy of the results. Therefore, this study demonstrates the effectiveness of these methods in achieving higher classification accuracy in monitoring the pilot’s mental workload. Specifically, this study achieves an accuracy of 86.4% using multinomial logistic regression, highlighting its reliability for this application. This study has some limitations. The first limitation of this study was that the author did not counterbalance the order of the scenario. All the pilots started in the low-load/pilot monitoring condition and then in the high-load/pilot flying condition. The second limitation of this work is that the author could not control for all the variables, such as wind conditions, as these experiments were conducted under realistic settings. The third limitation of this work is that one has to consider that the workload was not stationary in each leg of the flight pattern (namely, takeoff, crosswind, downwind, base, and final). However, the goal was not to compare each of these legs, especially as long as the duration of these legs is not equal, thus making it difficult to perform statistical comparisons across these legs without having the same number of data points. The authors believe that this approach is valid as long as the first and second traffic patterns include the same legs.

The final limitation is that the model’s performance was assessed using nine-fold cross-validation, which, while providing a robust estimate of classification accuracy, does not fully account for real-world generalization on unseen data. Since model parameters were selected based on cross-validation performance, there is a possibility of dataset-specific biases influencing the results. Future work will focus on evaluating the model with an independent, unseen dataset collected under real flight conditions to further assess its generalizability and practical applicability in aviation settings. Additionally, incorporating external validation with different pilot groups and flight conditions will strengthen the reliability of this approach.

## 5 Conclusion

This study demonstrates a high degree of accuracy in detecting a pilot’s mental workload using a six-dry-electrode EEG system under real flight conditions. This offers a promising method for monitoring the brain performance in realistic settings with only a few electrodes. By selecting important features and using an optimal classification method, accuracy can be improved while reducing computational costs and time. However, increasing the number of electrodes and using wet electrodes may not be practical for real flight conditions. In the next phase, mental workload estimation for both the pilot and co-pilot could help optimize task allocation based on workload distribution.

## Data Availability

The data analyzed in this study are subject to the following licenses/restrictions: The data are not publicly available but can be made available by requesting the corresponding author. Requests to access these datasets should be directed to noman.naseer@au.edu.pk.
